# RETRACTION: Internalization of *Staphylococcus aureus* in Lymphocytes Induces Oxidative Stress and DNA Fragmentation: Possible Ameliorative Role of Nanoconjugated Vancomycin

**DOI:** 10.1155/omcl/9803095

**Published:** 2026-07-15

**Authors:** Oxidative Medicine and Cellular Longevity

RETRACTION: S. P. Chakraborty, S. Kar Mahapatra, S. K. Sahu, et al., “Internalization of *Staphylococcus aureus* in Lymphocytes Induces Oxidative Stress and DNA Fragmentation: Possible Ameliorative Role of Nanoconjugated Vancomycin,” *Oxidative Medicine and Cellular Longevity* 2011, no. 1 (2011): 1–15, https://doi.org/10.1155/2011/942123.

The above article, published online on 18 September 2011 in Wiley Online Library (wileyonlinelibrary.com), has been retracted by agreement between the Journal Editor, Jeannette Vasquez‐Vivar and John Wiley & Sons Ltd.

The retraction has been agreed upon following an investigation into concerns raised by a third party and PubPeer comments [1] regarding image inconsistencies. Figure 15 shows evidence of the reuse of bands within the different conditions of VSSA and VSSA+Van, and distinct lines caused by the addition of bands to the image. The authors’ responses regarding these findings were unsatisfactory.

The authors were informed of the retraction, but did not respond.
